# Myeloid malignancies in the real-world: Occurrence, progression and survival in the UK’s population-based Haematological Malignancy Research Network 2004–15

**DOI:** 10.1016/j.canep.2016.03.011

**Published:** 2016-06

**Authors:** Eve Roman, Alex Smith, Simon Appleton, Simon Crouch, Richard Kelly, Sally Kinsey, Catherine Cargo, Russell Patmore

**Affiliations:** a Epidemiology and Cancer Statistics Group, Department of Health Sciences, University of York, YO10 5DD, UK; b St. James’s Institute of Oncology, Leeds Teaching Hospitals NHS Trust, LS9 7TF, UK; c Leeds General Infirmary, Leeds Teaching Hospitals NHS Trust, LS1 3EX, UK; d Queens Centre for Oncology, Castle Hill Hospital, HU16 5JQ, UK

**Keywords:** Myelodysplastic syndromes, Acute myeloid leukaemia, Myeloproliferative neoplasms, Polycythaemia vera, Essential thrombocythaemia

## Abstract

**Background:**

Population-based information on cancer incidence, prevalence and outcome are required to inform clinical practice and research; but contemporary data are lacking for many myeloid malignancy subtypes.

**Methods:**

Set within a socio-demographically representative UK population of ∼4 million, myeloid malignancy data (N = 5231 diagnoses) are from an established patient cohort. Information on incidence, survival (relative & overall), transformation/progression, and prevalence is presented for >20 subtypes.

**Results:**

The median diagnostic age was 72.4 years (InterQuartile Range 61.6–80.2), but there was considerable subtype heterogeneity, particularly among the acute myeloid leukaemias (AML) where medians ranged from 20.3 (IQR 13.9–43.8) for AML 11q23 through to 73.7 (IQR 57.3–79.1) for AML with no recurrent genetic changes. Five-year Relative Survival (RS) estimates varied hugely; from <5% for aggressive entities like therapy-related AML (2.6%, 95% Confidence Interval 0.4–9.0) to >85% for indolent/treatable conditions like chronic myeloid leukaemia (89.8%, 95% CI 84.0–93.6). With a couple of notable exceptions, males experienced higher rates and worse survival than females: the age-standardized incidence rates of several conditions was 2–4 higher in males than females, and the 5-year RS for all subtypes combined was 48.8% (95% CI 46.5–51.2) and 60.4% (95% CI 57.7–62.9) for males and females respectively. During follow-up (potential minimum 2 years and maximum 11 years) myelodysplastic syndrome (MDS) progression to AML ranged from 25% for refractory anaemia with excess blasts through to 5% for refractory anaemia with ring sideroblasts: the median interval between MDS and AML diagnosis was 9.0 months (IQR 4.8–17.4 months).

**Conclusions:**

The marked incidence and outcome variations seen by subtype, sex and age, confirm the requirement for “real-world” longitudinal data to inform aetiological hypotheses, healthcare planning, and future monitoring of therapeutic change. Several challenges for routine cancer registration were identified, including the need to link more effectively to diagnostic and clinical data sources, and to review policies on the recording of progressions and transformations.

## Highlights

• ICD-O3 analysis of “real-world” data revealed novel variations by sub-type, sex and age.
• Males experience higher incidence and worse survival than females; the reasons for this are unknown.
• Lack of concordance in the use of standard populations impacts markedly on the comparability of national incidence estimates.
• Lack of concordance in recording progressions impacts on the comparability of national AML occurrence & survival estimates.

## 1 Introduction

Accounting for around a third of all newly diagnosed haematological malignancies, myeloid neoplasms (acute myeloid leukaemias, myelodysplastic syndromes, and myeloproliferative neoplasms) comprise a complex group of cancers with diverse aetiologies, treatment pathways and outcomes [1], [2]. Contemporary population-based information about the occurrence and outcome for many of these malignancies is however sparse, and for some of the rarer cancer entities included within these categories is largely non-existent. This absence of relevant data reflects the paradigm changing nature of the new classification systems implemented over the last 15 years; the 2001 World Health Organization (WHO) schema for tumours of the haematopoietic and lymphoid tissue incorporating, for the first time, genetic data with information on morphology, immunology and clinical parameters [3]. This not only resulted in significant refinements to previously defined categories, but also to the addition of several new malignancies including, for example, the myelodysplastic syndromes (MDS) which are still assigned a morphology behaviour code of one and grouped with the ‘D codes’ in the latest update of the site-based International Statistical Classification of Diseases (ICD-10) [4]. Such radical changes posed significant problems for population-based cancer registries; many struggling to capture all haematological malignancies, particularly patients diagnosed with MDS and chronic myeloproliferative neoplasms [5], [6], [7] and continuing to report using the traditional ICD-10 groupings of leukaemia, Hodgkin lymphoma, non-Hodgkin lymphoma and myeloma for a number of years [8], [9], [10], [11].

In addition to the change in classification and breadth of investigations required to accurately diagnose haematological malignancies (histology, cytology, immunophenotyping, cytogenetics, flow cytometry and clinical data), a major factor impacting on routine cancer registration is the fact that unlike other cancers haematological malignancies are characterized by their ability to progress and transform [1], [2]. For example, certain MDS subtypes are, by their nature, disposed to progress to AML and, in order to deal with such phenomena, national and specialized cancer registries have applied a range of different policies [12], [13], [14], [15]. In 2010, the USA’s SEER (Surveillance, Epidemiology and End Results) program issued guidelines to allow primary AML registrations in patients with a prior MDS registration, unless the two diagnoses were ≤21 days apart [15]; the 2001 guidelines, which prohibited such registrations, having resulted in falling AML rates [16], [17], [18]. On the other hand, ENCR (European Network of Cancer Registries) and European HAEMCARE guidelines state that only the first tumour, in this example MDS, should be counted in incidence statistics, unless AML progression occurs within three months (90 days), in which case the original MDS code should be replaced by the appropriate AML code [13], [14]. As well as variations in case definition, making comparisons between the rates generated by different registries is further complicated by the fact that standard populations with widely differing age structures are often used for age-adjustment. European registries have, for example, generally used the 1976 European standard [19], [20], [21], [22], [23], [24], US SEER registries the US 2000 standard [16], [17], and registries from elsewhere in the world their own country specific standards and/or the 1996 World standard [25], [26], [27].

Since 2001, continued advances in genomics and diagnostic technologies have led to further WHO revisions, and haemato-oncology continues to be one of the most rapidly evolving fields in cancer research [1], [2]. Accordingly, to address the need for responsive “real-time” generalizable data on haematological malignancies to inform contemporary clinical practice and research, we established a population-based patient in cohort in the UK in 2004–the Haematological Malignancy Research Network (www.hmrn.org) [28]. Set within a catchment population of over 4 million people, all haematological malignancy diagnoses are made and coded by clinical specialists working at a single integrated haematopathology laboratory; and follow-up data are collected to clinical trial standards. Providing up-to-date information on patients diagnosed 2004–13 and followed through to September 2015, the present report focuses on the occurrence (incidence and prevalence) and outcomes (survival and transformations/progressions) across the full spectrum of myeloid neoplasms.

## 2 Methods

Data are from the UK’s population-based Haematological Malignancy Research Network (www.hmrn.org) which, with a catchment population of nearly 4 million people, has a socio-demographic composition that broadly mirrors that of the UK as a whole [29]. Initiated in 2004, full details of its structure, data collection methods and ethical approvals have been previously described [28]. Briefly, within HMRN patient care is provided by 14 hospitals organized into five multi-disciplinary teams (MDTs); and clinical practice adheres to national guidelines. As a matter of policy, all diagnoses, including progressions and transformations, are reported and coded by clinical haematopathology specialists at the Haematological Malignancy Diagnostic Service (www.hmds.info); this requirement occurs irrespective of the patient’s age, treatment intent, or management within the National Health Service (NHS) or private sector. HMDS, which is cited in the UK’s Department of Health guidance documents as the model for service delivery [30], [31], is a fully integrated facility; bringing together the relevant technology and expertise required for the diagnosis and on-going monitoring of all haematological malignancies. With respect to myeloid malignancies, the diagnosis of most remains centred on morphological assessment of the bone marrow; the subsequent integration of diagnostic characteristics with clinical features, in particular blood count parameters, enabling accurate diagnosis and subclassification. Importantly, within HMDS all bone marrows are dual reported to ensure accuracy, and a number of additional technologies are employed to confirm the diagnosis and refine classification, including cytogenetics and flow cytometry.

HMRN’s cohort has Section 251 support under the NHS Act 2006, and all patients have prognostic, treatment, response and outcome data collected to clinical trial standards; and all are ‘flagged’ and followed-up for death and subsequent cancer registrations at the national Medical Research Information Service (MRIS). For analytical purposes, area-based population counts are routinely sourced from the Office of National Statistics [32]. In the present report, all analyses were conducted either in the statistical package Stata 13 [33] or R [34]. Incidence rates and their 95% Confidence Intervals (CIs) were estimated by Poisson regression. Directly age-standardized rates were calculated using the Stata command ‘dstdize’ and corresponding age standardized sex rate ratios and their 95% confidence intervals were estimated [35]. Overall survival was calculated using standard time to event analyses and the program strel (v1.2.7) was used to estimate relative survival; age and sex-specific background mortality rates were obtained from national life tables [36]. Prevalence estimates (3-, 5- and 10-year) and corresponding confidence intervals were calculated from incidence and survival data using R’s ‘survival’ and ‘rms’ libraries; 3 and 5 year estimates were calculated directly from the patient cohort, and Monte-Carlo simulation techniques were employed to generate the larger 10-year values [37].

## 3 Results

Of the 5231 myeloid malignancies diagnosed September 2004 to August 2013, 4945 (94.5%) were new diagnoses (de novo) falling within one of the four main WHO diagnostic categories of acute myeloid leukaemia (AML), myelodysplastic syndromes (MDS), myeloproliferative neoplasms (MPN) and MDS/MPN; and 286 (5.5%) were secondary, following a previous diagnosis in another myeloid category. These data are distributed by gender and subtype (for those with more than 20 diagnoses) in Table 1. As expected, the largest difference between the total diagnostic series and the myeloid de novo series was seen for acute myeloid leukaemia (AML) with myelodysplasia related changes; 184 (93.4%) of the 197 diagnoses following a preceding myeloid malignancy. MDS accounted for 167 (90.8%) of the 184 prior diagnoses the median diagnostic interval between MDS and AML being 9.0 months (InterQuartile Range 4.8–17.4 months).

**Fig. 1:**
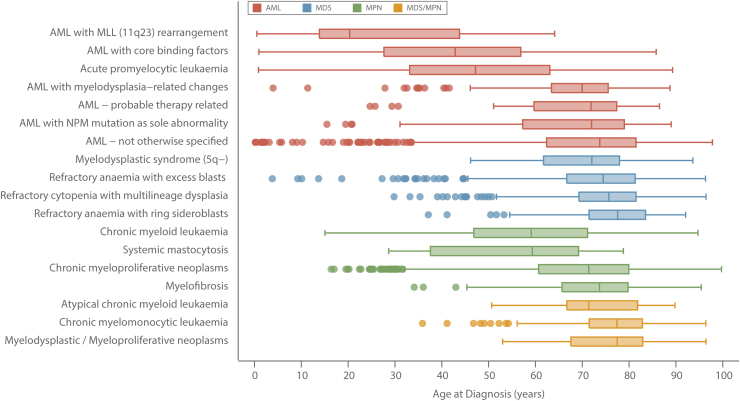
Age at diagnosis box and whisker plots by diagnostic group; acute myeloid leukaemias (AML), myelodysplastic syndromes (MDS), myeloproliferative neoplasms (MPN) and MDS/MPN: Haematological Malignancy Research Network diagnosed 2004–2013.

As with many other cancers, the likelihood of developing a myeloid malignancy increased markedly with increasing age (Table 2); the median age at diagnosis of all 5231 myeloid subtypes combined being 72.4 years (IQR 61.5–80.2 years) and, with relatively few exceptions, the patterns among males and females were broadly similar. However, as can be seen more clearly in the box and whiskers plots shown in Fig. 1, there is considerable variation both between and within the four WHO major groupings of AML, MDS, MPN, and MDS/MPN. This is particularly evident for AML (shown in red in Fig. 1), where the median diagnostic age ranged from 20.3 years (IQR 13.9–43.8 years) for patients diagnosed with AML that had an 11q23 rearrangement (N = 25) through to 73.7 years (IQR 62.3–81.7 years) for the larger group of patients that had AML with no recurrent genetic changes and was not therapy-related (N = 860). For most subtypes, the median diagnostic ages of those diagnosed with de novo disease was broadly comparable to that of the totality (Table 2).

The impact of the choice of standard population is clearly evident in Table 3, which shows the crude rates together with the age-adjusted rates calculated by applying our 5-year age-specific rates to four commonly used hypothetical standard populations (direct standardization). As might be expected, the new 2013 European Standard Population (ESP), which has the greatest weighting towards older ages, yielded age-adjusted summary rates that were closest to our own crude rates. By comparison, those produced using the 1996 world standard, the population with the greatest weighting towards younger groups, are approximately half the size. Furthermore, whilst those resulting from the widely used USA 2001 and European 1976 standards are more closely aligned, the older age distribution of the USA 2001 standard nonetheless yields rates that are consistently higher than those produced by the 1976 European standard.

**Fig. 2:**
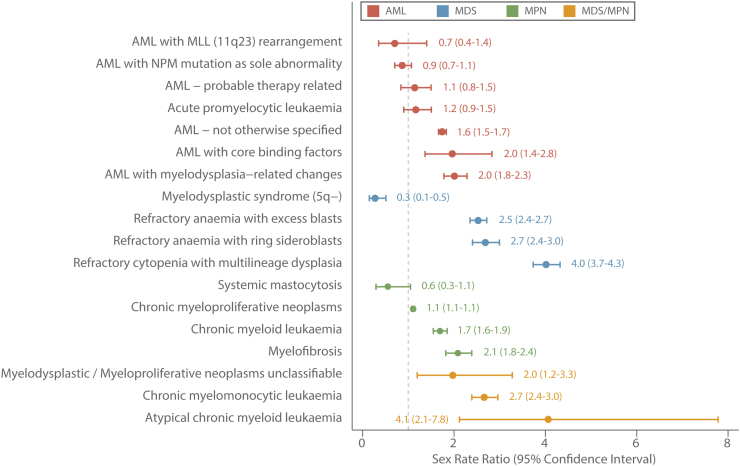
Age-standardized (European 2013) sex rate-ratios by diagnostic group; acute myeloid leukaemias (AML), myelodysplastic syndromes (MDS), myeloproliferative neoplasms (MPN) and MDS/MPN: Haematological Malignancy Research Network diagnosed 2004–2013.

In general, with a couple of notable exceptions, myeloid malignancies tend to occur far more frequently in males than females. These gender differences are plainly visible in Fig. 2, which shows the age-standardized (European 2013) sex-specific rate ratios (male rate/female rate) ordered by magnitude within each of the four main subtype groupings. As with age, there is variation both between and within the main diagnostic groups, the range for MDS being particularly marked; female patients having a significant predominance among those diagnosed with MDS that had an isolated 5q deletion (MDS 5q-; male rate/female rate = 0.27, 95% Confidence Interval 0.14–0.51) and males predominating in all other subtypes, the rate ratio for those with refractory cytopenia with multilineage dysplasia being the highest at 4.02 (95% CI 3.73–4.32).

Prevalence estimates (3-, 5-, and 10-year) based on all data are presented in Table 4. Data for 4 subtypes (MDS 5q-, systemic mastocytosis, MDS/MPN unclassified, and atypical CML) are not presented because of small numbers. For all myeloid malignancies, the combined prevalence ranged from 34.3 per 100,000 (95% CI 32.4–36.3 per 100,000) within 3 years of diagnosis, through to 50.0 per 100,000 (95% CI 47.7–52.4 per 100,000) within 5 years and 79.2 per 100,000 (95% CI 86.2–82.2 per 100,000) within 10 years. For some conditions, such as APL, the prevalent pools will contain individuals who have been cured of their cancer; the proportion increasing as time from diagnosis increases. For others, particularly the MPNs where the 3-, 5- and 10-year estimates per 100,000 are 21.7 (95% CI 20.2- 23.3), 33.2 (95% 31.3–35.0) and 56.8 (95% CI 54.2–59.3) respectively, the prevalent pools will contain individuals who are either being actively monitored or who are receiving treatment for their disease.

**Fig. 3:**
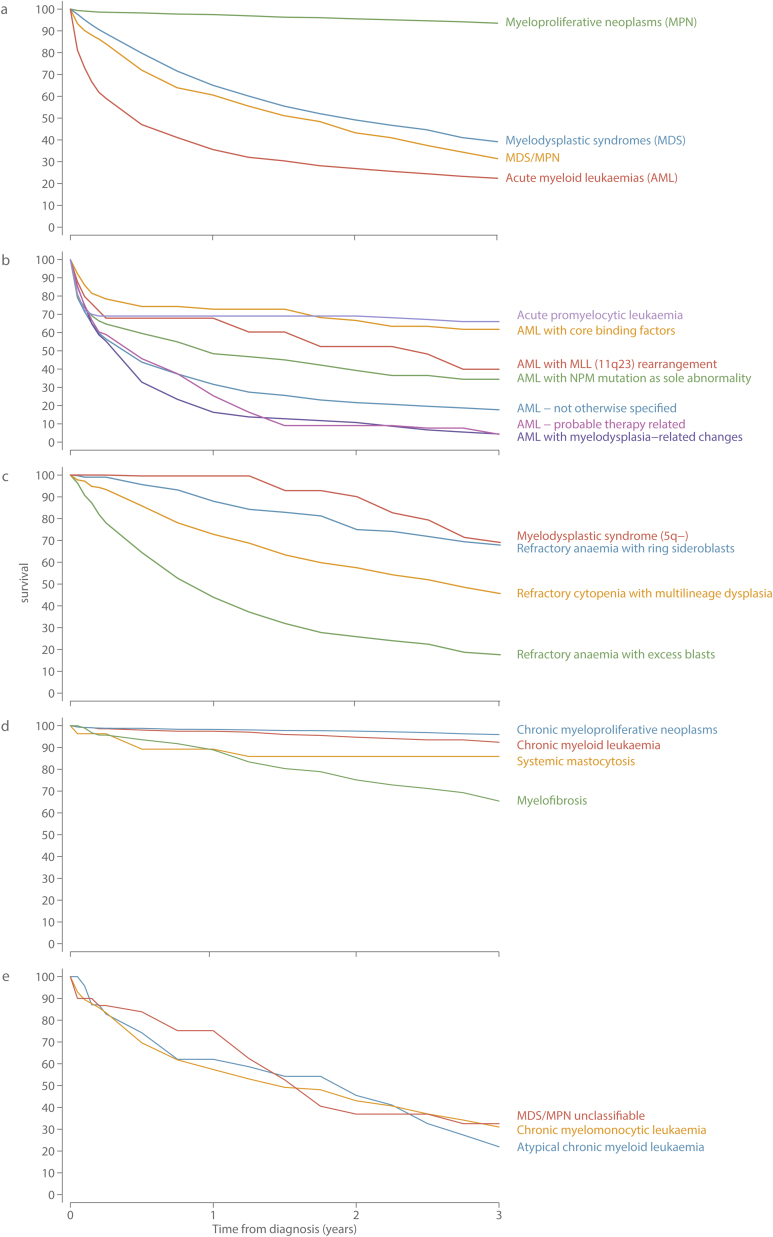
3-year relative survival estimates a) main diagnostic group; b) acute myeloid leukaemias (AML); c) myelodysplastic syndromes (MDS); d) myeloproliferative neoplasms (MPN); e) MDS/MPN: Haematological Malignancy Research Network diagnosed 2004–2013.

The aggressive nature of most myeloid malignancies is evident from the 5-year overall and relative survival (RS) estimates shown in Table 5, and the corresponding 3-year relative survival curves in Fig. 3; both Table and the Figure being based on all 5231 diagnoses. With a 5-year RS of only 14.7% (95% CI 12.9–16.7%), patients diagnosed with AML fared the worst; the RS curve falling steeply within the first few months of diagnosis (Fig. 3a). Within the AML group there is, however, considerable variation by subtype; therapy-related AML and AML with myelodysplasia related changes being almost universally and rapidly fatal, whereas patients diagnosed with acute promyelocytic leukaemia (APL) or AML with core-binding factor mutations were more likely than not to survive for 5 years or more (Table 5); the relative survival curves of both of these subtypes falling steeply within the first 3 months but levelling off thereafter (Fig. 3b).

**Fig. 4:**
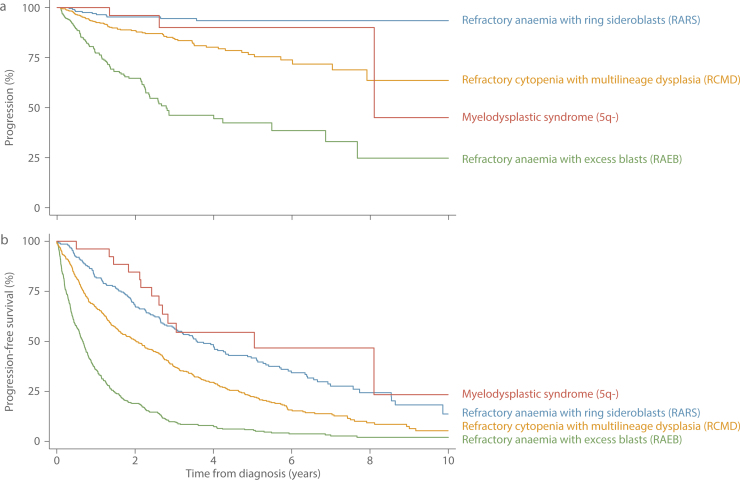
Myelodysplastic Syndromes (MDS) a) free from progression to AML b) progression free survival: Haematological Malignancy Research Network diagnoses Sept 2004 to Aug 2013, followed through to Feb 2015.

Overall, whilst outcomes for patients diagnosed with MDS are marginally better than those for patients diagnosed with AML (Fig. 3a), the 5-year RS of the MDS patient group as a whole, is only 28.1% (95% CI 24.9–31.5%) (Table 3). Furthermore, as with AML there is considerable heterogeneity across the various MDS entities (Fig. 3c), patients diagnosed with MDS 5q- faring considerably better (5-year RS = 68.7%; 95% CI 35.6–87.3%) than those with refractory anaemia and excess blasts (RAEB, 5-year RS = 9.9; 95% CI 6.9–13.6%). It is important to remember, however, that patients diagnosed with MDS that progressed to AML during the 9-year study period are currently counted in both Fig. 3b and c. The impact of this is illustrated more clearly in Fig. 4 where, in-line with mortality, the follow-up period for progression to AML has been extended by two years to September 2015. In total, 199 (16.6%) of the 1193 patients diagnosed with MDS between September 2004 and August 2013 had a subsequent diagnosis of AML before 1st September 2015. As expected, patients with RAEB were the most likely to progress; 116 (25%) of the 458 patients diagnosed with RAEB having a subsequent diagnosis of AML, the median time to progression being 9.3 months (IQR 4.5–19.4 months). Patients diagnosed with refractory cytopenia with multilineage dysplasia (RCMB) also exhibited comparatively high levels of progression; 13.9% (69/496) having a subsequent AML diagnosis, albeit over a longer time-frame (median time to progression = 15.2 months, IQR 6.9–33.6 months). In addition, although less impactful in terms of absolute numbers of diagnoses, patients with MDS 5q- and refractory anaemia with ring sideroblasts (RARS) also contributed to the both groups: the respective progression frequencies being 11.5% (n = 3/26; median time to progression = 31.8 months) and 5.2% (n = 11/213; median time to progression = 10.9 months). Finally, the progression free survival curves shown in Fig. 4b, confirm the generally poor outcomes for patients with all four MDS subtypes.

In stark contrast to other myeloid groups, the mortality experience of patients diagnosed with MPNs approached that of the general population (5-year RS 89.3%; 95% CI 86.9–91.3); the most favourable outcomes being seen for patients with chronic MPNs (5-year RS 93.1%, 95% CI 90.2–95.1%). JAK2 mutations have been used to diagnose all chronic MPNs within the study region since 2005; but the classification into the main component subtypes of polycythaemia vera (PV), essential thrombocythaemia (ET) and MPNs unclassified, which requires access to blood count and other clinical parameters, has not been routinely applied. However, as part of a clinical audit we assembled population-based information for a 60 month period (Sept 2006-Aug 2009, Sept 2011-Aug 2013), and the incidence rates per 100,000 (crude and age-adjusted) and 5-year OS and RS estimates are shown in Table 6. With a Standardized (European 2013) Incidence Rate of 4.35 (95%CI 4.2–4.5) per 100,000 ET is the commonest of the chronic MPNs, and those that were not further classifiable the rarest (SIR 0.79, 95%CI 0.72–0.87). The sex-rate ratios of ET (1.06) and PV (1.05) were similar, and 5-year RS estimates of both were over 90% (Table 6). By contrast, patients in the unclassifiable category had worse survival (5-year RS 75.9%; 95%CI 61.64–85.47%) and were more likely to be male (sex-rate ratio 1.94, 95%CI 1.58–2.37).

With a 5-year RS of only 42.0% (95% CI 31.5–52.1%), the 165 patients diagnosed with myelofibrosis stand apart from those with other MPNs (Fig. 3d). The survival of the comparatively small number of patients (n = 296) diagnosed with MDS/MPN disorders was uniformly poor (Table 5); the 5-year RS of the group as a whole being only 17.4% (95% CI 12.1–23.5%), with all three identified subtypes faring equally badly (Fig. 4e).

Lastly, within our population-based series the outcomes for females diagnosed with a myeloid malignancy tended to be marginally better than those of males (Table 5): the 5-year RS for all subtypes combined being 60.4% (95% CI 57.7–62.9%) and 48.8% (95% CI 46.3–51.2%) respectively (P < 0.001). Across MDS and MPN subtypes, this gender disparity appears to be of a fairly general nature, the 5-year RS estimates of all subtypes being lower for males than females, albeit not statistically significantly so. There is more heterogeneity amongst AML subtypes, most notably for AML with MLL (11q23) where the 5-year RS were 47.1% (16.0–72.9%) and 22.9% (5.9–46.5%) for males and females respectively. Interestingly AML (11q23) does not have a male predominance (Fig. 2), and onsets at a much younger age than other AML subtypes (Table 2).

## 4 Discussion

This paper presents ‘real-world’ contemporary data on incidence, prevalence, progression/transformation and survival across the myeloid malignancy spectrum; providing new information to inform aetiological hypotheses and plan health-care services, as well as supplying a much needed baseline from which to monitor the impact of future therapeutic changes. Our longitudinal approach enabled us to examine occurrence and outcome (death and progression) frequencies in the general patient population for the four main myeloid entities (AML, MDS, MPN and MDS/MPN), as well as for 18 constituent WHO defined subtypes. Our analyses not only evidenced the heterogeneity of this complex cancer group, but also uncovered a number of novel findings. For example, with respect to gender, our age-standardized rate-based analysis revealed much larger incidence differences between males and females than is generally thought to be the case [1], and our relative survival analysis showed that, in contrast to lymphoid subtypes [38], for most myeloid subtypes, outcomes for males are generally worse for males than females. In addition, our longitudinal examination of progressions and transformations, which explored the challenges such events present for routine cancer registration, highlighted key variations in policy that are currently impacting on national occurrence and survival estimates.

Major strengths of our study include its large well-defined population-based catchment area, completeness of case ascertainment, detailed follow-up and world-class diagnostics; all of which combine to ensure that HMRN’s patient cohort is not affected by the data quality issues commonly faced by many population-based cancer registries [5], [6], [7], [8], [9], [10], [11], [12], [13], [14], [15], [16], [17], [19], [26], [27]. With respect to diagnosis, as one of the largest integrated haematopathology laboratories in Europe the Haematological Malignancy Diagnostic Service (HMDS), which lies at the centre of HMRN, has a strong track-record of national/international research and diagnostic policy adheres to European guidelines (www.hmds.info). In accord with WHO and European recommendations [2], [39], [40], bone marrow evaluation is a mandatory requirement for all MDS and AML diagnoses, including transformations and progressions, and flow cytometry immunophenotyping is a core feature of the diagnostic pathway, along with cytogenetics and molecular studies. Nonetheless HMDS is subject to some of the same limitations as other diagnostic laboratories. The heavy reliance on morphology, for example, particularly in relation to the diagnosis of MDS, remains a problem due to poor inter-observer concordance and the numerous non-neoplastic conditions that can mimic MDS [41], [42]. For this reason, within HMDS patients with refractory cytopenia with unilineage dysplasia (RCUD) are not assigned a WHO ICD-O3 code at this point in their pathway, instead they are flagged for close clinical monitoring. The frequency of AML with myelodysplasia related changes is also likely to be an underestimate since HMDS only include patients with a previous or concurrent myelodysplastic diagnoses, and not those with poor cytogenetics.

Further diagnostic challenges are present for those categories that generally require access to clinical data as well as sample material. For example, in the sub-classification of therapy related myeloid conditions currently clinical data are only incorporated at HMDS for AML. In the future, however, additional information about preceding and succeeding cancers will be obtained via linkage to nationally compiled cancer registration and hospital episode statistics (HES); permitting a more in-depth analyses of second cancers and therapy related disorders across all haematological malignancy subtypes (myeloid and lymphoid). Likewise, at HMDS JAK2 mutations are used to diagnose chronic MPNs; but the further breakdown into polycythaemia vera (PV) and essential thrombocythaemia (ET) requires access to blood count data and other clinical parameters, and these procedures have only recently been routinely incorporated. Reliable data on chronic MPNs are, however sparse and the five years of incidence data presented in the present report, which lie towards the top end of the published ranges, add to the body of knowledge on this topic [43], [44].

Weighting to a common standard population is required in order to make incidence comparisons within and between populations; and because registries tend to use different standards we applied our rates to three commonly used hypothetical populations (European 1976, USA 2001, and World 1996), as well as to the new 2013 European standard which is set to form the basis of future European health care statistics. With respect to UK national reference comparisons, data are only published for AML (all subtypes combined) and CML; and in this context it is important to note that our age standardized (European 1976) incidence rates of 3.48 per 100,000 for AML and 0.89 per 100,000 CML are closely aligned to the similarly standardized rates of 3.40 and 0.89 per 100,000 reported for England as a whole [22]. In the USA, in addition to data on AML and CML, SEER publish population-based incidence estimates for chronic myelomonocytic leukaemia (CMML), and chronic myleoproliferative neoplasms, as well as MDS (all subtypes combined). With respect to CMML and chronic MPNs, our USA 2001 standardized rates of 0.60 and 4.74 per 100,000 are significantly higher than those reported by SEER; their 2010 rates being 0.42 and 2.61 per 100,000 respectively. For chronic MPNs (polycythaemia vera, essential thombocythemia and MPN-unclassified) the difference is most likely due to the comparatively benign nature of these diseases and the consequent failure to capture all diagnoses within SEER [45], [46], whereas for CMML misscategorization to CML is the more likely cause [1]. For MDS, however, SEER’s 2010 overall rate of 5.31 per 100,000 (http://seer.cancer.gov/faststats/) is greater than our equivalently standardized (USA 2001) rate of 3.07 per 100,000. This difference largely reflects the fact that instead of assigning a final diagnosis of refractory cytopenia with unilineage dysplasia (RCUD), which in specialist European MDS registries accounts for around 10–20% of MDS diagnoses [1], [20], [21], [47], [48], [49], HMDS report these patients and flag them for follow-up. Furthermore, in contrast to many other registries the fully integrated nature of HMDS’s reporting system means that the category “MDS not otherwise specified (NOS)” is neither used nor needed; and so our rates for the more clearly defined MDS subtypes of refractory cytopenia with multilineage dysplasia (RCMD), refractory anaemia with excess blasts (RAEB), refectory anaemia with ring sideroblasts (RARS), and MDS 5q- tend to be higher than those produced by MDS registries that obtain data from multiple sources [6], [20], [21], [27], [48], [50].

With respect to progression and transformation frequencies, the levels documented thus far during our follow-up period (minimum 2 years, maximum 11 years) are broadly in line with those reported by other specialist registries [1], [2], [51]: MDS to AML progression ranging, for example, from 25% for RAEB and 14% RCMD, through to 12% for MDS 5q- and 5% for RARS. Unfortunately as with the use of standard populations, the policies applied by population-based cancer registries to document progressions and transformations differ one from another; and such variations impact on the incidence and survival statistics produced. The European Network of Cancer Registry (ENCR) guidelines state, for example, that if AML is diagnosed within 90 days (3 months) of an initial MDS diagnosis, the MDS diagnosis should be changed to AML and no record of the transformation need be kept; but if AML is diagnosed after 90 days the transformation should, if possible, be noted but only the MDS should be counted in incidence estimates [14]. In our data, 171 (86.0%) of the 199 MDS to AML transformations (diagnosed 09/04 to 08/13, followed-up to 09/15) occurred after 90 days (our maximum interval was 8.1 years; median 11.5 months); and so under ENCR guidelines these AMLs would not be counted. However, in contrast to ENCR, SEER’s guidelines specify that unless the diagnoses of MDS and AML are ≤ 21 days apart (our minimum interval was 32 days) both should be counted in incidence estimates [15]. Hence, our approach is basically similar to SEER’s and that used in a Swedish analysis of AML cancer registration data [12], and accords with WHO’s coding rules which assign AML patients with a previous MDS diagnosis to the “AML with myelodysplasia-related changes” category [1].

Contemporary real-world population-based information on the survival of patients diagnosed with myeloid malignancies are exceedingly sparse since, as with incidence, data on the categories defined in WHO’s 2001 diagnostic revision have only been published for some AML subtypes [12], [52], [53], [54], some MDS subtypes [21], [51], [53], [54], and CML [23], [53], [54], [55], [56], [57]. Hence our comprehensive up-to-date analysis of WHO defined subtypes is a major contribution to the literature which, as far as we can tell, has not been replicated elsewhere. Importantly such information provides the context for interpretation of data from clinical trials, as well as the baseline against which to evaluate the impact of new therapeutic advances across the patient population as a whole [58]. Furthermore, the fact that our subtype survival estimates are broadly consistent with those that have been published on by others [12], [21], [23], [51], [52], provides further evidence [59] that patients diagnosed with haematological malignancies in the UK do not suffer from the survival inequalities commonly reported for cancers, such as breast, colorectum and lung [60].

With respect to gender differences, our analyses not only confirm the large, but so far unexplained, fact that compared to females males are at significantly increased risk of developing most myeloid subtypes, but also highlighted consistent disparities in survival: the 5-year relative survival for all myeloid malignancies combined being significantly lower in males (48.8%; 95% CI 46.3–51.2) than females (60.4%; 95% CI 57.7–62.9). With respect to incidence, the consistency of the male excess, which reached four-fold for RCMD and atypical CML, is striking; and also serve to highlight subtypes that failed to exhibit such differences. In this regard, with its well-known distinct female excess [1], [51] yielding a sex-rate ratio of 0.3 (95% 0.1–0.5) in our data, MDS 5q- stands apart from other MDS subtypes. Interestingly, within our patient cohort incidence rates of most lymphoid subtypes are also significantly higher among males than females although, in contrast to the myeloid malignancies reported on here, no differences in outcome were evident [38].

In summary, our contemporary longitudinal analysis of “real-world” population-based data on myeloid malignancies categorised by WHO subtype demonstrated marked incidence and survival variations by subtype, age and sex; providing valuable base-line information not only for researchers, clinicians and patients, but also for service commissioners and regulators. In addition, we also identified some key challenges for routine cancer registration; the lack of concordance on the recording of progressions/transformations, which impacts on both incidence and survival estimates, being one such issue deserving the attention of policy makers.

## Authorship contribution

ER, AS, and RP were responsible for the conception and design of the study. AS, SA and SC carried out all of the analyses. CC, RK, SK and RP provided clinical input regarding the collection data and the analysis, as well as interpretation of the findings. ER and AS are the study guarantors and take responsibility for the integrity of the data. All authors contributed to the final draft of the paper; and have had full access to all of the data in the study.

## Conflict of interest

None of the authors have any conflicts of interest

## Figures and Tables

**Table 1:** Total numbers of myeloid diagnoses and de Novo diagnoses: HMRN Sept 2004 to Aug 2013.

	Diagnoses		Males		Females	
Malignancy (International Classification of Disease for Oncology 3rd Edition)	Total	Myeloid de novo (% of total)	Total	Myeloid de novo (% of total)	Total	Myeloid de novo (% of total)
All myeloid malignancies	5231	4945 (94.5)	2868	2691 (93.8)	2363	2254 (95.4)

Acute myeloid leukaemia (AML) (9727, 9861, 9871, 9866, 9895, 9896, 9920)	1411	1190 (84.3)	769	631 (82.1)	642	559 (87.1)
AML, not otherwise specified (9861)	860	825 (95.9)	475	452 (95.2)	385	373 (96.9)
AML with myelodysplasia-related changes (9895)	197	13 (6.6)	121	7 (5.8)	76	6 (7.9)
AML with NPM1 mutation (9861)	104	104 (100.0)	42	42 (100.0)	62	62 (100.0)
Acute promyelocytic leukaemia (APL) (9866)	91	91 (100.0)	47	47 (100.0)	44	44 (100.0)
AML, core binding factor (9871, 9896)	64	64 (100.0)	41	41 (100.0)	23	23 (100.0)
AML, probable therapy related (9920)	61	59 (96.7)	28	27 (96.4)	33	32 (97.0)
AML with MLL (11q23) (9897)	25	25 (100.0)	10	10 (100.0)	15	15 (100.0)

Myelodysplastic syndromes (MDS) (9982–9986)	1194	1188 (99.5)	794	790 (99.5)	400	398 (99.5)
Refractory cytopenia with multilineage dysplasia (RCMD) (9985)	497	495 (99.6)	364	362 (99.5)	133	133 (100.0)
Refractory anaemia with excess blasts (RAEB) (9983)	458	455 (99.3)	291	290 (99.7)	167	165 (98.8)
Refractory anaemia with ring sideroblasts (RARS) (9982)	213	212 (99.5)	135	134 (99.3)	78	78 (100.0)
Myelodysplastic syndrome (5q-) (9986)	26	26 (100.0)	4	4 (100.0)	22	22 (100.0)

Myeloproliferative neoplasms (MPN) (9741, 9875, 9950, 9961, 9962, 9964, 9975. 9875)	2330	2296 (98.5)	1118	1100 (98.4)	1212	1196 (98.7)
Chronic MPNs^a^ (9950, 9962, 9975)	1819	1812 (99.6)	820	815 (99.4)	999	997 (99.8)
Chronic myeloid leukaemia (CML) (9875)	318	316 (99.4)	189	188 (99.5)	129	128 (99.2)
Myelofibrosis (9961)	165	140 (84.8)	99	87 (87.9)	66	53 (80.3)
Systemic mastocytosis (9741)	26	26 (100.0)	8	8 (100.0)	18	18 (100.0)

MDS/MPN (9945, 9946, 9975, 9876)	296	271 (91.6)	187	170 (90.9)	109	101 (92.7)
Chronic myelomonocytic leukaemia (CMML) (9945)	239	221 (92.5)	152	140 (92.1)	87	81 (93.1)
MDS/MPN, unclassifiable (9975)	30	24 (80.0)	17	13 (76.5)	13	11 (84.6)
Atypical chronic myeloid leukaemia (9876)	23	22 (95.7)	17	16 (94.1)	6	6 (100.0)

a Polycythaemia vera, essential thrombocythaemia, MPNs unclassified.

**Table 2:** Median ages (Inter Quartile Range-IQR) of patients diagnosed with myeloid malignancies: HMRN 2004 to Aug 2013.

	All patients		Males		Females	
	Total N = 5231	Myeloid de novo N = 4945	Total N = 2879	Myeloid de novo N = 2691	Total N = 2378	Myeloid de novo N = 2254
All myeloid malignancies	72.4 (61.6–80.2)	72.5 (61.3–80.4)	72.0 (61.8–79.6)	72.1 (61.5–79.8)	72.7 (61.2–81.1)	72.9 (60.9–81.4)

Acute myeloid leukaemia(AML)	70.6 (57.3–79.1)	70.9 (55.4–79.6)	69.8 (57.4–78.2)	69.8(55.3–78.4)	71.2 (57.2–80.4)	71.7 (56.5–81.0)
AML, not otherwise specified	73.7 (62.4–81.5)	73.7 (62.3–81.7)	72.4 (61.7–79.9)	72.4(61.5–80.0)	75.8 (64.3–84.0)	75.8 (64.0–84.0)
AML with myelodysplasia-related changes	70.0 (63.4–75.5)	77.0 (72.2–78.2)	70.1 (63.6–75.3)	75.1(72.2–78.0)	69.9 (62.4–76.8)	77.6 (73.5–78.6)
AML with NPM1 mutation	72.0 (57.3–79.0)	72.0 (57.6–79.0)	70.6 (50.3–79.5)	70.6(50.3–79.5)	72.3 (60.0–78.9)	72.3 (60.0–78.9)
Acute promyelocytic leukaemia(APL)	47.2 (33.1–63.1)	47.2 (33.1–63.1)	48.0 (33.1–63.1)	48.0(33.1–63.1)	47.1 (32.8–60.9)	47.1 (32.8–60.9)
AML, core binding factor	42.9 (27.6–56.9)		41.8(28.4–57.2)		44.3(24.6–54.6)	
AML, probable therapy related	71.9 (59.7–77.4)	72.4 (59.5–78.4)	72.6 (67.7–76.6)	72.7(66.8–77.4)	68.6 (58.6–78.4)	67.5(58.6–78.5)
AML with MLL(11q23)	20.3 (13.9–43.8)		29.2(13.9–44.6)		20.3(13.2–39.4)	

Myelodysplastic syndromes(MDS)	75.7 (68.5–81.7)	75.7(68.5–81.7)	75.7(68.5–81.4)	75.8(68.6–81.4)	75.6(68.5–82.6)	75.7(68.5–82.6)
Refractory cytopenia with multilineage dysplasia (RCMD)	75.7 (69.3–81.5)	75.7(69.3–81.5)	75.7(69.4–81.2)	75.7(69.6–81.2)	75.9(68.5–82.5)	
Refractory anaemia with excess blasts (RAEB)	74.5 (66.7–81.3)	74.6(66.8–81.5)	74.9(67.2–81.0)	75.0(67.4–81.0)	73.6(65.9–81.9)	74.2(65.9–81.9)
Refractory anaemia with ring sideroblasts (RARS)	77.6 (71.4–83.5)	77.5(71.4–83.5)	76.9(69.6–83.0)	76.9(69.6–82.3)	78.9(72.1–83.7)	78.9(72.1–83.7)
Myelodysplastic syndrome (5q-)	72.0(61.7–78.0)		78.6(70.2–84.0)		69.6(61.4–77.3)	

Myeloproliferative neoplasms (MPN)	70.3(58.5–79.2)	70.2(58.4–79.2)	68.2(57.6–77.8)	68.1(57.6–77.8)	71.7(59.9–80.4)	71.7(59.7–80.3)
Chronic MPNs^a^	71.4(60.7–79.9)	71.4(60.6–79.9)	69.7(60.0–78.8)	69.6(59.8–78.8)	72.5(61.7–81.1)	72.5(61.7–81.1)
Chronic myeloid leukaemia (CML)	59.1(46.8–71.1)	59.1(46.8–71.1)	57.7(46.7–69.5)	57.8(46.7–69.5)	61.3(47.7–73.1)	61.2(47.3–73.0)
Myelofibrosis	73.7(65.7–79.8)	74.1(65.3–80.0)	72.0(63.4–79.0)	72.8(63.4–79.1)	75.4(68.4–81.9)	75.6(68.4–81.9)
Systemic mastocytosis	59.3(37.6–69.2)		70.6(66.2–72.2)		48.3(34.6–59.9)	

MDS/MPN	77.2(69.4–82.8)	77.4(70.7–83.1)	76.3(69.4–82.0)	76.4(69.4–82.4)	77.8(70.0–83.5)	78.4(71.6–84.2)
Chronic myelomonocytic leukaemia	77.4(71.5–82.9)	77.4(71.6–83.1)	76.4(69.9–82.0)	76.4(71.3–82.1)	78.4(72.5–83.5)	78.9(73.4–84.2)
MDS/MPN, unclassifiable	77.5(67.6–82.9)	78.4(71.3–84.1)	77.2(67.6–82.4)	78.4(71.7–82.9)	77.7(68.0–85.0)	78.4(71.0–86.4)
Atypical chronic myeloid leukaemia	71.4(66.7–81.8)	72.0(66.7–81.8)	71.4(68.2–81.8)	73.2(67.8–82.5)	68.1(55.3–79.8)	

a Polycythaemia vera, essential thrombocythaemia, MPNs unclassified.

**Table 3:** Crude and age standardized incidence rates per 100,000 (95% confidence interval): HMRN Sept 2004 to Aug 2013.

	Crude	European 2013	European 1976	USA 2001	World 1996
All myeloid malignancies	16.28 (15.84–16.72)	19.06 (18.89–19.23)	12.41 (12.29–12.52)	13.64 (13.51–13.76)	8.76 (8.58–8.67)
Acute myeloid leukaemia	4.39 (4.16–4.63)	5.06 (4.97–5.15)	3.48 (3.41–3.54)	3.75 (3.69–3.82)	2.58 (2.52–2.63)
AML-not otherwise specified	2.68 (2.50–2.86)	3.13 (3.06–3.20)	2.00 (1.95–2.05)	2.24 (2.19–2.29)	1.41 (1.37–1.46)
AML with myelodysplasia-related changes	0.61 (0.53–0.70)	0.73 (0.69–0.76)	0.49 (0.46–0.52)	0.50 (0.48–0.53)	0.35 (0.32–0.37)
AML with NPM mutation	0.32 (0.26–0.39)	0.37 (0.34–0.40)	0.25 (0.23–0.28)	0.28 (0.25–0.30)	0.18 (0.16–0.20)
Acute promyelocytic leukaemia	0.28 (0.23–0.35)	0.30 (0.27–0.33)	0.27 (0.25–0.30)	0.27 (0.25–0.30)	0.24 (0.21–0.27)
AML, core binding factor	0.20 (0.15–0.25)	0.20 (0.18–0.23)	0.20 (0.17–0.22)	0.20 (0.17–0.22)	0.18 (0.16–0.21)
AML, probable therapy related	0.19 (0.15–0.24)	0.22 (0.20–0.25)	0.15 (0.13–0.17)	0.16 (0.14–0.18)	0.11 (0.09–0.12)
AML with MLL(11q23)	0.08 (0.05–0.11)	0.07 (0.05–0.09)	0.09 (0.06–0.11)	0.08 (0.06–0.10)	0.10 (0.07–0.12)
Myelodysplastic syndromes (MDS)	3.72 (3.51–3.93)	4.44 (4.35–4.52)	2.58 (2.53–2.64)	3.01 (2.95–3.07)	1.67 (1.63–1.71)
Refractory cytopenia with multilineage dysplasia	1.55 (1.41–1.69)	1.85 (1.80–1.91)	1.07 (1.03–1.10)	1.25 (1.21–1.29)	0.68 (0.65–0.70)
Refractory anaemia with excess blasts	1.43 (1.30–1.56)	1.69 (1.64–1.74)	1.02 (0.98–1.06)	1.16 (1.12–1.20)	0.68 (0.65–0.71)
Refractory anaemia with ring sideroblasts	0.66 (0.58–0.76)	0.79 (0.76–0.83)	0.43 (0.41–0.46)	0.53 (0.50–0.56)	0.27 (0.25–0.28)
MDS (5q-)	0.08 (0.05–0.12)	0.10 (0.07–0.12)	0.06 (0.05–0.08)	0.07 (0.05–0.08)	0.04 (0.03–0.05)
Myeloproliferative neoplasms (MPN)	7.25 (6.96–7.55)	8.47 (8.35–8.58)	5.73 (5.64–5.81)	6.13 (6.05–6.22)	4.02 (3.96–4.09)
Chronic MPNs	5.66 (5.40–5.93)	6.65 (6.55–6.76)	4.37 (4.30–4.44)	4.74 (4.66–4.81)	3.02 (2.97–3.07)
Chronic myeloid leukaemia	0.99 (0.88–1.10)	1.10 (1.06–1.14)	0.89 (0.86–0.93)	0.90 (0.86–0.93)	0.69 (0.66–0.72)
Myelofibrosis	0.51 (0.44–0.60)	0.61 (0.58–0.65)	0.38 (0.35–0.40)	0.42 (0.39–0.44)	0.25 (0.23–0.27)
Systemic mastocytosis	0.08 (0.05–0.12)	0.09 (0.06–0.12)	0.08 (0.05–0.10)	0.07 (0.05–0.09)	0.06 (0.04–0.08)
MDS/MPN	0.92 (0.82–1.03)	1.10 (1.06–1.15)	0.62 (0.59–0.65)	0.74 (0.71–0.78)	0.40 (0.38–0.43)
Chronic myelomonocytic leukaemia	0.74 (0.65–0.84)	0.89 (0.85–0.93)	0.49 (0.46–0.52)	0.60 (0.57–0.63)	0.30(0.28–0.32)
MDS/MPN, unclassified	0.09 (0.06–0.13)	0.11 (0.09–0.14)	0.06 (0.05–0.08)	0.07 (0.06–0.09)	0.04 (0.03–0.05)
Atypical chronic myeloid leukaemia	0.07 (0.05–0.11)	0.09 (0.07–0.11)	0.05 (0.04–0.07)	0.06 (0.04–0.07)	0.04 (0.03–0.05)

**Males**					
All myeloid malignancies	18.42 (17.75–19.11)	25.14 (24.82–25.45)	15.63 (15.44–15.83)	17.7 (17.7–17.92)	10.76 (10.48–10.62)
Acute myeloid leukaemia	4.94 (4.60–5.30)	6.50 (6.34–6.66)	4.29 (4.19–4.40)	4.74 (4.62–4.86)	3.11 (3.02–3.20)
AML-not otherwise specified	3.05 (2.78–3.34)	4.17 (4.04–4.30)	2.60 (2.51–2.69)	2.95 (2.86–3.05)	1.80 (1.73–1.88)
AML with myelodysplasia-related changes	0.78 (0.64–0.93)	1.02 (0.95–1.10)	0.67 (0.61–0.72)	0.70 (0.64–0.76)	0.46 (0.42–0.50)
AML with NPM mutation	0.27 (0.19–0.36)	0.35 (0.28–0.42)	0.23 (0.18–0.28)	0.27 (0.21–0.32)	0.16 (0.12–0.20)
Acute promyelocytic leukaemia	0.30 (0.22–0.40)	0.32 (0.26–0.38)	0.29 (0.24–0.34)	0.29 (0.24–0.34)	0.25 (0.20–0.30)
AML, core binding factor	0.26 (0.19–0.36)	0.28 (0.22–0.34)	0.26 (0.21–0.31)	0.26 (0.21–0.32)	0.24 (0.18–0.29)
AML, probable therapy related	0.18 (0.12–0.26)	0.25 (0.19–0.30)	0.15 (0.11–0.19)	0.26 (0.21–0.32)	0.10 (0.7–0.14)
AML with MLL(11q23)	0.06 (0.03–0.12)	0.06 (0.03–0.09)	0.07 (0.03–0.11)	0.17 (0.13–0.21)	0.08 (0.03–0.12)
Myelodysplastic syndromes (MDS)	5.10 (4.75–5.47)	7.41 (7.24–7.59)	4.10 (4.00–4.20)	4.98 (4.86–5.10)	2.55 (2.48–2.62)
Refractory cytopenia with multilineage dysplasia	2.34 (2.10–2.59)	3.41 (3.29–3.53)	1.87 (1.80–1.94)	2.28 (2.20–2.36)	1.15 (1.11–1.20)
Refractory anaemia with excess blasts	1.87 (1.66–2.10)	2.68 (2.57–2.79)	1.52 (1.46–1.59)	1.82 (1.74–1.89)	0.97 (0.92–1.01)
Refractory anaemia with ring sideroblasts	0.87 (0.73–1.03)	1.28 (1.21–1.36)	0.69 (0.64–0.73)	0.86 (0.80–0.91)	0.42 (0.39–0.45)
MDS (5q-)	0.03 (0.01–0.07)	0.04 (0.01–0.07)	0.02 (0.00–0.04)	0.03 (0.00–0.05)	0.01 (0.00–0.02)
Myeloproliferative neoplasms (MPN)	7.18 (6.77–7.61)	9.45 (9.26–9.64)	6.28 (6.16–6.41)	6.79 (6.65–6.92)	4.37 (4.28–4.46)
Chronic MPNs	5.27 (4.91–5.64)	7.08 (6.91–7.25)	4.55 (4.45–4.66)	4.99 (4.87–5.11)	3.10 (3.02–3.19)
Chronic myeloid leukaemia	1.21 (1.05–1.40)	1.41 (1.33–1.49)	1.14 (1.07–1.20)	1.15 (1.08–1.21)	0.87 (0.82–0.93)
Myelofibrosis	0.64 (0.52–0.77)	0.88 (0.81–0.95)	0.54 (0.49–0.58)	0.59 (0.54–0.64)	0.35 (0.32–0.38)
Systemic mastocytosis	0.05 (0.02–0.10)	0.06 (0.03–0.10)	0.04 (0.02–0.07)	0.04 (0.02–0.07)	0.03 (0.01–0.05)
MDS/MPN	1.20 (1.04–1.39)	1.78 (1.69–1.88)	0.96 (0.90–1.02)	1.19 (1.12–1.26)	0.59 (0.54–0.64)
Chronic myelomonocytic leukaemia	0.98 (0.83–1.14)	1.45 (1.37–1.54)	0.77 (0.72–0.83)	0.97 (0.91–1.03)	0.47 (0.43–0.50)
MDS/MPN, unclassified	0.11 (0.06–0.17)	0.16 (0.11–0.21)	0.09 (0.06–0.12)	0.11 (0.07–0.14)	0.05 (0.03–0.07)
Atypical chronic myeloid leukaemia	0.11 (0.06–0.17)	0.16 (0.11–0.21)	0.09 (0.06–0.12)	0.10 (0.07–0.13)	0.06 (0.04–0.08)

**Females**					
All myeloid malignancies	14.26 (13.69–14.85)	15.07 (14.87–15.28)	10.10 (9.95–10.25)	10.96 (10.81–11.11)	7.33 (7.09–7.21)
Acute myeloid leukaemia	3.87 (3.58–4.19)	4.07 (3.96–4.18)	2.86 (2.78–2.95)	2.16 (2.08–2.23)	3.06 (2.98–3.15)
AML-not otherwise specified	2.32 (2.10–2.57)	2.41 (2.32–2.50)	1.55 (1.48–1.62)	1.74 (1.67–1.81)	1.10 (1.04–1.16)
AML with myelodysplasia-related changes	0.46 (0.36–0.57)	0.51 (0.45–0.56)	0.36 (0.31–0.40)	0.36 (0.31–0.40)	0.26 (0.21–0.30)
AML with NPM mutation	0.37 (0.29–0.48)	0.40 (0.35–0.46)	0.28 (0.23–0.32)	0.29 (0.25–0.34)	0.20 (0.16–0.24)
Acute promyelocytic leukaemia	0.27 (0.19–0.36)	0.28 (0.23–0.33)	0.26 (0.22–0.31)	0.26 (0.21–0.31)	0.23 (0.18–0.27)
AML, core binding factor	0.14 (0.09–0.21)	0.14 (0.10–0.19)	0.14 (0.10–0.18)	0.14 (0.10–0.18)	0.13 (0.09–0.18)
AML, probable therapy related	0.20 (0.14–0.28)	0.22 (0.18–0.26)	0.16 (0.13–0.19)	0.16 (0.12–0.19)	0.11 (0.08–0.14)
AML with MLL(11q23)	0.09 (0.05–0.15)	0.09 (0.05–0.12)	0.10 (0.06–0.14)	0.10 (0.06–0.14)	0.12 (0.07–0.16)
Myelodysplastic syndromes (MDS)	2.41 (2.18–2.66)	2.53 (2.44–2.62)	1.54 (1.48–1.61)	1.75 (1.68–1.82)	1.04 (0.98–1.10)
Refractory cytopenia with multilineage dysplasia	0.80 (0.67–0.95)	0.85 (0.79–0.90)	0.51 (0.47–0.55)	0.58 (0.54–0.62)	0.33 (0.30–0.37)
Refractory anaemia with excess blasts	1.01 (0.86–1.17)	1.06 (0.99–1.13)	0.68 (0.62–0.73)	0.75 (0.69–0.80)	0.48 (0.42–0.53)
Refractory anaemia with ring sideroblasts	0.47 (0.37–0.59)	0.48 (0.43–0.52)	0.26 (0.23–0.29)	0.32 (0.29–0.35)	0.16 (0.14–0.18)
MDS (5q-)	0.13 (0.08–0.20)	0.15 (0.11–0.19)	0.10 (0.07–0.13)	0.10 (0.07–0.13)	0.07 (0.05–0.09)
Myeloproliferative neoplasms (MPN)	7.31 (6.91–7.74)	7.79 (7.64–7.94)	5.30 (5.19–5.41)	5.68 (5.57–5.79)	3.75 (3.67–3.84)
Chronic MPNs	6.03 (5.66–6.41)	6.42 (6.28–6.55)	4.27 (4.17–4.36)	4.61 (4.51–4.71)	2.98 (2.90–3.05)
Chronic myeloid leukaemia	0.78 (0.65–0.93)	0.83 (0.78–0.89)	0.67 (0.62–0.72)	0.68 (0.63–0.73)	0.52 (0.48–0.57)
Myelofibrosis	0.40 (0.31–0.51)	0.42 (0.37–0.47)	0.25 (0.22–0.29)	0.29 (0.25–0.32)	0.16 (0.14–0.19)
Systemic mastocytosis	0.11 (0.06–0.17)	0.12 (0.08–0.16)	0.11 (0.07–0.15)	0.10 (0.07–0.14)	0.09 (0.06–0.12)
MDS/MPN	0.66 (0.54–0.79)	0.68 (0.62–0.73)	0.40 (0.35–0.44)	0.47 (0.42–0.51)	0.27 (0.23–0.31)
Chronic myelomonocytic leukaemia	0.53 (0.42–0.65)	0.54 (0.49–0.59)	0.29 (0.26–0.33)	0.36 (0.32–0.40)	0.18 (0.16–0.21)
MDS/MPN, unclassifiable	0.08 (0.04–0.13)	0.08 (0.05–0.11)	0.05 (0.03–0.07)	0.05 (0.03–0.08)	0.03 (0.02–0.04)
Atypical chronic myeloid leukaemia	0.04 (0.01–0.08)	0.04 (0.01–0.07)	0.03 (0.00–0.05)	0.03 (0.01–0.05)	0.02 (0.00–0.04)

**Table 4:** 3-, 5-and 10-year prevalence estimates (95% confidence intervals) per 100,000 of myeloid malignancies: Haematological Malignancy Research Network diagnoses Sept 2004 to Aug 2013, followed through to Feb 2015.

	Total			Males			Females		
	3 Year	5 Year	10 Year	3 Year	5 Year	10 Year	3 Year	5 Year	10 Year
All myeloid malignancies	34.3(32.4–36.3)	50.0(47.7–52.4)	79.2(86.2–82.2)	37.7(34.8–40.6)	55.4(51.9–58.9)	82.6(78.3–87.0)	31.2(28.6–33.7)	45.0(42.0–8.1)	76.0(72.0–80.0)

Acute myeloid leukaemia	4.4(3.7–5.1)	6.0(5.2–6.8)	9.0(8.0–10.0)	5.1(4.1–6.2)	7.1(5.9–8.4)	10.4(8.9–11.9)	3.7(2.8–4.6)	4.9(3.9–5.9)	7.6(6.4–8.9)
AML-not otherwise specified	2.1(1.6–2.6)	2.7(2.1–3.2)	3.8(3.1–4.4)	2.7(1.9–3.5)	3.6(2.7–4.5)	4.9(3.8–5.9)	1.5(1.0–2.1)	1.8(1.2–2.4)	2.8(2.0–3.6)
AML with myelodysplasia related changes	0.5(0.1–0.8)	0.6(0.3–1.0)	0.6(0.3–1.0)	0.4(0.1–0.7)	0.5(0.2–0.8)	0.5(0.2–0.8)	0.4(0.2–0.6)	0.6(0.3-–0.8)	0.6(0.3–0.8)
AML with NPM mutation	0.6(0.4–0.9)	1.0(0.6–1.3)	1.2(0.8–1.6)	0.8(0.3–1.2)	0.9(0.4–1.3)	1.1(0.6–1.6)	0.5(0.2–0.8)	1.0(0.6–1.5)	1.3(0.7–1.8)
Acute promyelocytic leukaemia	0.6(0.4–0.9)	0.9(0.6–1.2)	1.8(1.4–2.3)	0.6(0.2–0.9)	0.9(0.5–1.4)	2.0(1.3–2.6)	0.7(0.3–1.1)	0.9(0.4–1.3)	1.7(1.1–2.3)
AML, probable therapy related	0.1(0.0–0.3)	0.2(0.0–0.3)	0.2(0.0–0.3)	0.1(0.0–0.3)	0.2(0.0–0.4)	0.2(0.0–0.4)	0.2(0.0–0.3)	0.2(0.0–0.3)	0.2(0.0–0.4)
AML with core binding factors	0.4(0.2–0.6)	0.6(0.3–0.8)	1.3(0.8–1.6)	0.4(0.1–0.7)	0.8(0.3–1.2)	1.4(0.8–1.9)	0.3(0.1–0.6)	0.4(0.1–0.7)	1.1(0.6–1.5)
AML with MLL(11q23)	0.1(0.0–0.2)	0.1(0.0–0.2)	0.(0.1–0.5)	0.1(0.0–0.3)	0.1(0.0–0.3)	0.4(0.1–0.6)	0.1(0.0–0.3)	0.1(0.0–0.3)	0.3(0.0–0.5)

Myelodysplastic syndromes (MDS)	6.3(5.5–7.1)	8.7(7.7–9.6)	10.9(9.8–12.0)	8.7(7.3–10.1)	11.7(10.1–13.3)	14.4(12.6–16.2)	4.0(3.1–4.9)	5.8(4.7–6.9)	7.5(6.3–8.8)
Refractory cytopenia with multilineage dysplasia	3.1(2.5–3.6)	4.2(3.5–4.8)	5.1(4.3–5.9)	4.7(3.7–5.7)	6.4(5.2–7.6)	7.7(6.4–9.0)	1.5(1.0–2.1)	2.1(1.4–2.7)	2.7(1.9–3.4)
Refractory anaemia with excess blasts	1.7(1.2–2.1)	2.0(1.5–2.4)	2.3(1.8–2.8)	2.3(1.6–3.0)	2.7(1.9–3.4)	3.0(2.1–3.8)	1.0(0.6–1.5)	1.3(0.8–1.8)	1.7(1.1–2.3)
Refractory anaemia with ring sideroblasts	1.3(0.9–1.7)	2.1(1.6–2.6)	3.0(2.4–3.5)	1.6(1.0–2.1)	2.5(1.8–3.3)	3.6(2.7–4.5)	1.0(0.6–1.5)	1.7(1.1–2.3)	2.3(1.6–3.0)

Myeloproliferative neoplasms (MPN)	21.7(20.2–23.3)	33.2(31.3–35.0)	56.8(54.2–59.3)	21.6(19.4–23.8)	33.9(31.2–36.7)	54.1(51.3–58.3)	21.8(19.7–24.0)	32.4(29.8–35.0)	58.6(55.1–62.1)
Chronic MPNs^a^	16.8(15.4–18.1)	25.7(24.0–27.3)	44.4(42.2–46.6)	16.4(14.5–18.3)	25.6(23.2–27.9)	41.1(38.0–44.1)	17.1(15.2–19.0)	25.8(23.5–28.1)	47.6(44.4–50.7)
Chronic myeloid leukaemia	3.2(2.6–3.7)	4.7(4.0–5.4)	8.5(7.5–9.5)	3.6(2.7–4.5)	5.6(4.5–6.7)	10.1(8.6–11.7)	2.8(2.0–3.5)	3.8(2.9–4.7)	6.9(5.7–8.1)
Myelofibrosis	1.5(1.1–1.9)	2.0(1.5–2.5)	2.5(1.9–3.0)	1.5(0.9–2.1)	2.1(1.4–2.8)	2.5(1.8–3.3)	1.4(0.9–2.0)	1.8(1.2–2.5)	2.4(1.7–3.1)

MDS/MPN	1.9(1.5–2.4)	2.3(1.8–2.8)	2.6(2.0–3.1)	2.3(1.5–3.0)	2.6(1.8–3.4)	2.9(2.1–3.7)	1.6(1.0–2.2)	2.0(1.3–2.6)	2.3(1.6–3.0)
Chronic myelomonocytic leukaemia	1.2(0.8–1.6)	1.5(1.1–1.9)	1.7(1.3–2.2)	1.4(0.9–2.0)	1.8(1.2–2.4)	2.0(1.3–2.6)	1.0(0.5–1.4)	1.2(0.7–1.8)	1.5(0.9–2.1)

a Polycythaemia vera, essential thrombocythaemia, MPNs unclassified.

**Table 5:** Five-year overall survival (OS) and relative survival (RS) estimates (95% confidence interval) for myeloid malignancies: Haematological Malignancy Research Network diagnoses Sept 2004 to Aug 2013, followed through to September 2015.

	Total diagnoses		Males		Females	
	OS (95% CI)	RS (95% CI)	OS (95% CI)	RS (95% CI)	OS (95% CI)	RS (95% CI)
All myeloid malignancies	40.3 (38.9–41.7)	51.2 (49.5–52.9)	38 (36.0–40.0)	48.8 (46.3–51.2)	48.2 (45.9–50.4)	60.4 (57.7–62.9)

Acute myeloid leukaemia	12.9 (11.3–14.7)	14.7 (12.9–16.7)	12.8 (10.6–15.2)	14.7 (12.2–17.4)	13.3 (10.9–15.9)	14.9 (12.3–17.9)
AML, not otherwise specified	8.2 (6.6–10.0)	9.5 (7.7–11.7)	8.8 (6.5–11.4)	10.2 (7.6–13.3)	7.4 (5.2–9.9)	8.7 (6.2–11.7)
AML with myelodysplasia-related changes	2.8 (1.3–5.4)	3.1 (1.4–5.9)	1.8 (0.5–4.6)	2.0 (0.6–5.0)	4.6 (1.6–10.4)	4.9 (1.6–11.1)
AML with NPM mutation	22.2 (14.5–30.9)	25.0 (16.3–34.6)	25.3 (13.1–39.4)	29.0 (14.8–44.8)	21.0 (11.8–32.0)	23.4 (13.1–35.5)
Acute promyelocytic leukaemia	58.6 (47.0–68.4)	61.7 (49.4–71.9)	55.3 (38.8–69.0)	59.7 (41.4–74.0)	61.4 (44.5–74.6)	62.9 (45.4–76.2)
AML, core binding factor	55.3 (42.0–66.7)	57.1 (43.3–68.7)	50.0 (33.5–64.3)	51.2 (34.3–65.8)	64.3 (41.2–80.3)	66.8 (42.2–82.8)
AML, probable therapy related	2.4 (0.4–8.3)	2.6 (0.4–9.0)	5.2 (0.9–15.5)	5.80 (1.0–17.0)	1.1 (0.0–8.9)	1.2 (0.0–9.2)
AML with MLL (11q23)	31.1 (14.0–50.0)	31.4 (14.1–50.4)	46.7 (16.0–72.9)	47.1 (16.0–73.4)	22.8 (5.9–46.3)	22.9 (5.9–46.5)

Myelodysplastic syndromes (MDS)	21.2 (18.7–23.8)	28.1 (24.9–31.5)	19.2 (16.2–22.4)	25.5 (21.5–29.7)	24.8 (20.3–29.6)	32.0 (26.1–37.9)
Refractory cytopenia with multilineage dysplasia	23.1 (19.1–27.3)	31.1 (25.7–36.6)	21.2 (16.6–26.2)	28.4 (22.2–35.0)	27.3 (19.3–36)	36.4 (25.6–47.2)
Refractory anaemia with excess blasts	7.9 (5.5–10.9)	9.9 (6.9–13.6)	7.6 (4.5–11.8)	9.8 (5.7–15.1)	8.4 (4.6–13.8)	10.2 (5.5–16.6)
Refractory anaemia with ring sideroblasts	41.3 (34.2–48.3)	57.2 (47.1–66)	37.5 (28.6–46.3)	51.6 (39–62.8)	47.8 (35.0–59.5)	62.8 (45.0–76.2)
Myelodysplastic syndrome(5q-)	53.7 (31.6–71.4)	68.7 (35.6–87.3)	23.8 (0.8–65.5)	29.4 (0.6–74.7)	56.4 (31.1–75.5)	72.5 (31.9–91.4)

Myeloproliferative neoplasms (MPN)	69.7 (67.7–71.7)	89.3 (86.9–91.3)	68.0 (64.8–70.9)	87.6 (83.8–90.5)	73.2 (70.3–75.9)	92.8 (89.4–95.2)
Chronic MPNs^b^	71.5 (69.2–73.7)	93.1 (90.2–95.1)	70.0 (66.3–73.3)	93.9 (88.5–96.8)	73.8 (70.6–76.7)	94.3 (90.2–96.7)
Chronic myeloid leukaemia	77.7 (72.3–82.2)	89.8 (84.0–93.6)	78.3 (71.0–84.0)	88.7 (80.4–93.6)	77.0 (67.6–84.0)	92.1 (78.4–97.2)
Myelofibrosis	32.1 (24.2–40.2)	42.0 (31.5–52.1)	25.7 (16.3–36.3)	32.8 (20.5–45.7)	44.9 (28.3–60.2)	59.7 (35.8–77.1)
Systemic mastocytosis	79.8 (57.9–91.1)	88.7 (53.2–97.8)	59.5 (19.8–84.7)	62.9 (19.1–87.8)	87.9 (59.5–96.9)	95.5 (10.8–99.9)

MDS/MPN	13.0 (9.1–17.6)	17.4 (12.1–23.5)	9.4 (5.1–15.3)	12.6 (6.8–20.2)	19.7 (11.2–29.9)	26.8 (15.1–39.9)
Chronic myelomonocytic leukaemia	13.3 (9.1–18.4)	18.1 (12.3–24.8)	10.6 (5.7–17.4)	14.3 (7.5–23.1)	19.5 (10.6–30.5)	27.0 (14.4–41.3)
MDS/MPN, unclassified^b^	0	0	0	0	0	0
Atypical chronic myeloid leukaemia	0.2 (0.0–11.0)	0.2 (0.0–12.5)	0.1 (0.0–8.1)	0.1 (0.0–9.2)	17.4 (1.1–50.6)	18.5 (1.1–53.0)

^a^Polycythaemia vera, essential thrombocythaemia, MPNs unclassified.

b All 23 patients died within 5 years of diagnosis.

**Table 6:** Median age (Inter Quartile Range) Incidence rates (crude and age-standardized), sex-rate ratios, and 5-year overall survival (OS) and relative survival (RS) estimates (95% Confidence intervals) for polycythaemia vera, essential thrombocythaemia, and myleoproliferative neoplasms unclassified: Haematological Malignancy Research Network diagnoses Sept 2006 to Aug 2009, Sept 2011 to Aug 2013, followed through to September 2015.

		Polycythaemia vera			Essential thrombocythaemia			Myleoproliferative (MPN) Unclassifiable		
		All patients	Males	Females	All patients	Males	Females	All patients	Males	Females
Total diagnoses		310	140	170	663	289	374	120	70	50
Median age (IQR)		70.5 (61.4 −78.6)	65.3 (58.8–76.4)	73.1 (65.4–80.9)	71.5 (60.5–80.8)	71.0 (60.3–80.0)	72.2 (60.6–81.8)	72.3 (64.2–80.1)	72.3 (63.0–80.1)	73.8 (65.6–81.7)
Crude rate per 100,000		1.74 (1.55–1.94)	1.62 (1.36–1.91)	1.85 (1.58–2.15)	3.71 (3.44–4.01)	3.34 (2.97–3.75)	4.06 (3.66–4.5)	0.67 (0.56–0.8)	0.81 (0.63–1.02)	0.54 (0.40–0.72)
Age-standardized rates per 100,000	European 2013	2.06 (1.95–2.16)	2.09 (1.93–2.25)	1.99 (1.84–2.13)	4.35 (4.20–4.50)	4.57 (4.33–4.82)	4.31(4.11–4.51)	0.79 (0.72–0.87)	1.12 (0.98–1.26)	0.58 (0.49–0.66)
European 1976	1.36 (1.29–1.44)	1.44 (1.33–1.56)	1.27 (1.17–1.37)	2.84 (2.74–2.94)	2.85 (2.70–3.00)	2.91 (2.76–3.05)	0.5 (0.45–0.56)	0.69 (0.59–0.79)	0.36 (0.30–0.42)
USA	1.44 (1.37–1.52)	1.48 (1.37–1.60)	1.38 (1.28–1.48)	3.13 (3.02–3.24)	3.24 (3.07–3.41)	3.14 (2.99–3.29)	0.55 (0.50–0.61)	0.78 (0.67–0.89)	0.40 (0.33–0.46)
World	0.93 (0.88–0.98)	1.00 (0.92–1.09)	0.85 (0.78–0.93)	1.97 (1.89–2.04)	1.92 (1.81–2.03)	2.04 (1.93–2.16)	0.35 (0.31–0.39)	0.48 (0.4–0.56)	0.25 (0.20–0.29)
Male/female age-standardized (European 2013) sex rate ratio		1.05 (0.95–1.17)			1.06 (0.99–1.14)			1.94 (1.58–2.37)		
5-year overall survival%		77.68 (72.10–82.27)	76.71 (68.02–83.33)	78.49 (70.74–84.40)	71.19 (67.11–74.86)	68.41 (61.99–73.98)	73.34 (67.90–78.02)	57.98 (47.67–66.96)	61.25 (47.90–72.15)	54.26 (37.93–68.01)
5-year relative survival%		94.88 (87.46–97.96)	95.11 (77.92–99.0)	95.16 (81.37–98.81)	93.55 (87.95–96.60)	92.55 (81.40–97.13)	93.84 (85.86–97.38)	75.91 (61.64–85.47)	81.01 (59.83–91.73)	68.68 (46.32–83.24)
